# All-Metal Metamaterial-Based Sensor with Novel Geometry and Enhanced Sensing Capability at Terahertz Frequency

**DOI:** 10.3390/s25020507

**Published:** 2025-01-16

**Authors:** Sagnik Banerjee, Ishani Ghosh, Carlo Santini, Fabio Mangini, Rocco Citroni, Fabrizio Frezza

**Affiliations:** 1 Department of Information Engineering, Electronics and Telecommunications (DIET), “La Sapienza” University of Rome, 00184 Rome, Italy; banerjee.2055414@studenti.uniroma1.it (S.B.); carlo.santini@uniroma1.it (C.S.); fabio.mangini@uniroma1.it (F.M.); rocco.citroni@uniroma1.it (R.C.); 2 Department of Mechanical and Aerospace Engineering, “La Sapienza” University of Rome, 00184 Rome, Italy; ghosh.2046512@studenti.uniroma1.it

**Keywords:** all-metal, metamaterial, absorber, refractive index, sensitivity

## Abstract

This research proposes an all-metal metamaterial-based absorber with a novel geometry capable of refractive index sensing in the terahertz (THz) range. The structure consists of four concentric diamond-shaped gold resonators on the top of a gold metal plate; the resonators increase in height by 2 µm moving from the outer to the inner resonators, making the design distinctive. This novel configuration has played a very significant role in achieving multiple ultra-narrow resonant absorption peaks that produce very high sensitivity when employed as a refractive index sensor. Numerical simulations demonstrate that it can achieve six significant ultra-narrow absorption peaks within the frequency range of 5 to 8 THz. The sensor has a maximum absorptivity of 99.98% at 6.97 THz. The proposed absorber also produces very high-quality factors at each resonance. The average sensitivity is 7.57/Refractive Index Unit (THz/RIU), which is significantly high when compared to the current state of the art. This high sensitivity is instrumental in detecting smaller traces of samples that have very correlated refractive indices, like several harmful gases. Hence, the proposed metamaterial-based sensor can be used as a potential gas detector at terahertz frequency. Furthermore, the structure proves to be polarization-insensitive and produces a stable absorption response when the angle of incidence is increased up to 60°. At terahertz wavelength, the proposed design can be used for any value of the aforementioned angles, targeting THz spectroscopy-based biomolecular fingerprint detection and energy harvesting applications.

## 1. Introduction

Metamaterials are purposefully made composite structures that are designed to have unique properties not present in any other naturally occurring material, such as a negative refractive index, negative permittivity, and optical magnetism [1]. Negative permittivity usually occurs when the effective permittivity shows a negative behavior with respect to the frequency. Usually, metals at optical frequencies depict such behavior, referred to, in scientific terms, as plasmonic [2]. On the other hand, negative refractive index behavior is exhibited when both the effective permittivity and permeability depict negative magnitudes as a function of frequency; these materials are termed left-handed materials [3]. Metamaterial absorbers (MMAs) absorb electromagnetic radiation and transform it into heat. They are often used in energy harvesting [4], absorption modulation [5], and sensor and detecting systems [6]. MMAs are an increasingly important research topic comprising multi-layer metal-dielectric structures with a conductive resonator, dielectric spacer, and metal ground plane [7,8,9,10,11,12]. Recent research has focused on developing and investigating terahertz MMAs [13,14,15,16,17,18,19,20,21,22,23,24]. Adjusting the geometry, dimension, and electromagnetic properties of MMA unit-cell structures on a sub-wavelength scale can result in optimal absorption across many bands [13,14,15,16,17,18,19]. MMAs have limited practical uses due to their complicated geometrical structural configurations and time-consuming manufacturing techniques.

Channel estimation is a promising application of all-metal technology, especially for 6G terahertz (THz) antennas. A cost-effective THz multibeam antenna has been developed using magnetron sputtering and surface Pμ SL 3D printing techniques [20]. Future sixth-generation (6G) wireless networks will heavily depend on sensing to support emerging scenarios such as Industry 4.0, smart cities, and smart homes within the Internet of Everything (IoE). These applications require not only high data rate transmission but also high-resolution and precise sensing capabilities, which are central to the integrated sensing and communications (ISAC) paradigm.

The THz frequency bands offer extensive spectral resources that can significantly enhance ISAC’s communication and sensing functions. Moreover, massive MIMO (m-MIMO) [21] technology, which is pivotal for 5G and future 6G networks, can provide substantial array gain to counteract the high propagation losses in THz bands, thereby improving communication capacity and spatial resolution for sensing.

The integration of THz, m-MIMO, and ISAC is expected to deliver ultra-broad bandwidth and numerous spatial degrees of freedom, facilitating ultra-high resolution target detection and rapid communication transmission. Recent studies have addressed target sensing and channel estimation challenges, proposing effective techniques for THz-m-MIMO-ISAC systems that utilize channel training to estimate target parameters. Notably, the fusion of ISAC and THz m-MIMO enhances spectrum and energy efficiency by allowing the sharing of hardware and spectral resources for both communication and sensing tasks.

Recently, there has been increased exploration of THz frequencies for sensing and imaging applications. THz sensors hold great promise for a variety of uses, particularly in label-free sensing of micro-samples, such as gas traces and cancer cells, due to their non-invasive probing capabilities. This manuscript explores these aspects in detail from an electromagnetic perspective.

To meet the demand for simple and small MMA designs, three alternative techniques have been implemented to improve THz wave absorption performance [25,26,27,28,29,30,31,32]. The first case is a high-index dielectric resonator combined with a ground metal plane [27,28]. This effectively absorbs terahertz waves through the use of hybrid dielectric guide resonance or Mie resonance. The second case is using a structural semiconductor material as an all-dielectric resonator [29,30]. This allows for the narrow-band, broadband, and adjustable absorption of THz waves. The last case is a three-dimensional all-metal resonator [31,32] that achieves narrowband perfect absorption and improves the application of the refractive index. A micro-ring-shaped structure composed of a GaAs array with a golden ground plane and dielectric substrate showed an absorption of 99.9% at 2.21 THz [28]. The InSb-based thermo-sensitive semiconductor material was used as a substrate to produce a metasurface absorber, which produces a narrow-band absorption of 99.9% at 1.89 THz [30]. An all-metal structure formed with copper achieved an absorption of more than 99.99% [32].

THz sensing technology, in conjunction with metamaterials, offers the advantages of high sensitivity, label-free operation, and enhanced electromagnetic wave–analyte interactions. The emergence of various synthetic materials has led to an increase in the potential for THz spectroscopy-based biomolecular fingerprint detection with high sensitivity. The exotic electromagnetic properties exhibited by metamaterials have shown more promising results and fields of application at the THz frequency spectrum. Metamaterials have the potential to greatly increase THz-sensor sensitivity. Split ring and patch resonators can be used to create metamaterial characteristics: [33] demonstrates a structure consisting of a ground plane and a bottom layer made up of gold, along with a dielectric substrate made up of gallium arsenide. Although the absorption level of this structure is 99.5%, the sensitivity of the sensor is 5.16 Refractive Index Unit (THz/RIU), whereas, in the proposed design, higher sensitivities are obtained at all the six peaks with an average of 7.57 THz/RIU [34]. In [2], several circular ring resonators composed of gold are designed over a gallium arsenide substrate; the structure achieves an absorption of 99%, but the sensitivity of the sensor remains 1.447 THz/RIU. In [35], a similar structure composed of circular ring resonators made up of aluminum on the top of a GaAs substrate shows an absorption of 99.5% but only 1.5 THz/RIU sensitivity. Hence, it is apparent that conventional triple-layered metamaterials, typically consisting of a metallic ground plane, a sandwiched dielectric substrate, and a top metal plane, are not excellent choices as sensors due to poor sensitivity. To address this problem, all-metal metamaterials have been proposed [36,37,38,39,40,41,42,43,44,45], providing relatively higher sensitivities when employed as sensors. When it comes to sensing analytes in which the refractive indices are very closely placed or are highly co-related, achieving a still-higher sensitivity and a multi-band performance remains a challenge. Sensitivities as high as 197.49 THz/RIU or 68.65 THz/RIU in all-metal structures have been reported in the relevant literature [36,38] but are still very difficult to achieve physically in THz spectroscopy applications, considering the current state of the art. Nowadays, non-invasive probing of analytes is currently possible even within the previously unexplored range between 0.1 THz and 10 THz, commonly referred to as the “terahertz gap”.

This paper depicts a design of an all-metal metamaterial absorber that exhibits six significant absorption peaks in the THz spectrum. The novelty lies in the fact that a peculiar non-planar geometry has been proposed that aids the sensing capabilities, thereby employing the absorber as a refractive index sensor. The proposed design consists of an all-metal gold structure of multiple resonating rings arranged in a novel distinctive geometry, in which the height of the ring resonators is increased in steps of 2 µm, proceeding from the outer to the inner ones. The outstanding performance of this new design is represented by its hexa-narrowband near-perfect absorption: the structure produces six absorption peaks, with the highest peak being at 6.97 THz with 99.98% absorption. Results of simulations, reported in the polarization angle plot, confirm that the structure is polarization insensitive up to 90° of normal incidence. At terahertz frequencies, the developed metamaterial-based sensor offers improved sensing capabilities, proved by a sensitivity of 5.67 THz/RIU, 5.1 THz/RIU, 7.45 THz/RIU, 7.92 THz/RIU, 7.25 THz/RIU, and 11.03 THz/RIU, in the six resonance peaks, respectively, in the frequency range between 5.972 THz and 7.934 THz. Perfect absorption at six different frequencies and enhanced sensing capabilities at terahertz frequency represent unique and outstanding features of the new design. To further strengthen our above proposition, in Table 1, we present a comparison of the proposed metamaterial-based sensor with the current state of the art according to various important performance metrics.

The paper is organized as follows: in Section 2, we describe the geometrical and structural features of the unit cell and the configuration of the numerical simulation software employed for its electromagnetic study. In Section 3, we present the results of simulations on absorption capabilities and resonance of the proposed structure, justifying our conclusions on the metamaterial nature of our design. Eventually, in Section 4, we present the conclusions of the paper and describe the limitations of our study, future developments, and possible applications.

## 2. Materials and Methods

**Figure 1:**
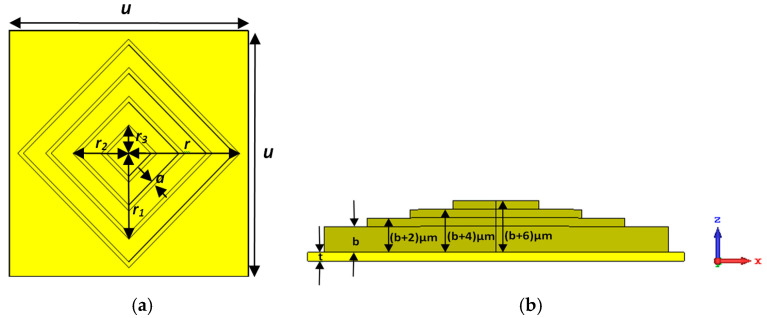
(**a**) Front view of the proposed design. (**b**) Side view of the proposed design. Geometrical dimensions of the structure are u = 86 µm, r = 40 µm, b = 6 µm, r_1_ = 30 µm, r_2_ = 20 µm, and r_3_ = 10 µm, respectively. a = 2 µm, b = 6 µm, and t = 2 µm.

Figure 1 reports a depiction of the unit cell composing the all-metal metamaterial absorber. On the top of a gold 2 µm thick metal plate, four concentric square resonators with parallel sides are positioned. The resonators are made of gold with a conductivity of 4.56 × 10^7^ Sm^−1^. The complete structure is obtained by replicating the unit cell in the orthogonal direction along the diagonals of the square resonators: the unit cell periodicity (*u*) measures 86 µm. The circumradius (*r*) of the largest square measures 40 µm, while the square ring’s thickness and height (*b*) measure 2 µm and 6 µm, respectively. Likewise, the circumradii of the remaining three resonators are 30 µm (*r*_1_), 20 µm (*r*_2_), and 10 µm (*r*_3_), respectively. All the square ring resonator has the same thickness (*a*) of 2 µm. The outermost square ring’s height (*b*) measures 6 µm; the inner second, third, and fourth square ring resonators have heights of 8 µm, 10 µm, and 12 µm, respectively. For the reader’s convenience, values of geometrical dimensions are reported in Table 2. In the proposed design, the inner resonator rings are higher than the outer ones, giving the structure a step-pyramidal shape when viewed from the side, resulting in improved performance compared to other sensors reported in the recent literature.

Drude’s model characterizes the optical characteristics of noble metals, such as gold, using the real and imaginary parts of a complex permittivity [41].

(1) $$\varepsilon_{h}\left(\omega \right)=\varepsilon_{h}-\frac{\omega_{m}^{2}}{\omega^{2}+j\omega \gamma }$$

where *ω* is the operating angular frequency (rad/s), $\varepsilon_{h}\;$ is the high-frequency permittivity (12.94), *ω_m_* is the plasma angular frequency, and *γ* is the damping constant. The plasma angular frequency can be expressed by the equation [42]:

(2) $$\omega_{m}={\left(\frac{Ne^{2}}{m\varepsilon_{0}}\right)}^{\frac{1}{2}}$$

where *N* is the electron density, *e* is the electron charge, *m* is the electron mass, and *ε*_0_ is the free-space permittivity.

All the numerical simulations were run using the commercial software CST Microwave Studio. The mentioned software to solve Maxwell’s Equations in the frequency domain uses the finite integration technique. This approach produces a discrete frequency response with constant steps over the whole bandwidth. The frequency domain solver is a very helpful tool able to retrieve electromagnetic wave responses in any material at any frequency value while testing electrically smaller constructions. By using tetrahedral meshing, periodic boundary conditions are imposed on both sides of the unit cell. By solving electromagnetic wave propagation along the *z*-axis, effects on the top surface of the sensor are analyzed.

## 3. Results and Discussions

**Figure 2:**
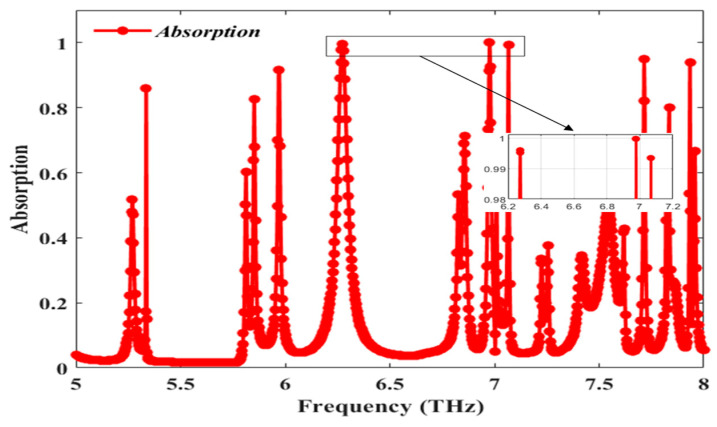
Absorption spectra of the proposed structure with hexaband configuration.

The calculation of the absorbance of the structure involves deducting the total transmittance and reflectivity from unity (1). Furthermore, $T_{w}={\left|S_{21}\right|}^{2}$ and $R_{w}={\left|S_{11}\right|}^{2}$, which represent the transmission and reflection coefficients, respectively. The transmittance of this proposed structure is zero due to the presence of the metal plate, which acts as the ground plane. Since the skin depth of gold is greater than the wavelength of incident radiation, the excitation wave sees the metal plate as an infinite medium and cannot escape from the material. The new equation becomes $A_{w}=1-R_{w}$. Minimal reflection and transmission are needed to attain perfect absorption. Since this structure allows zero transmission, the reflection coefficient should be nearly equal to zero to grant perfect absorption. This can be obtained by impedance matching the surface of the absorber with that of free space (377 Ω). Figure 2 portrays six perfect absorption peaks of 91.52% at 5.97 THz, 99.6% at 6.27 THz, 99.98% at 6.97 THz, 99.34% at 7.07 THz, 94.85% at 7.71 THz, and 93.82% at 7.93 THz, respectively. Table 3 reports values of calculated parameters used to assess the performance of the proposed design.

**Figure 3:**
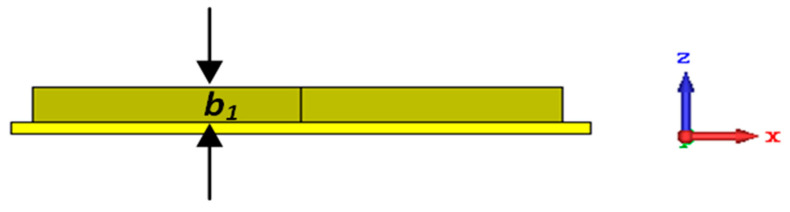
Side view of a conventional sensor [31,32].

The novelty of the proposed structure is apparent when compared to conventional designs of all-metal absorbers reported in the relevant literature [38,39,40,41,42,43]. The THz absorber proposed in [31,32] (represented in Figure 3)—although based on a unit cell with a similar footprint and geometrical dimensions compared to our proposed one—is nevertheless composed of four square concentric resonators of the same height (*b*_1_) = 6 µm.

**Figure 4:**
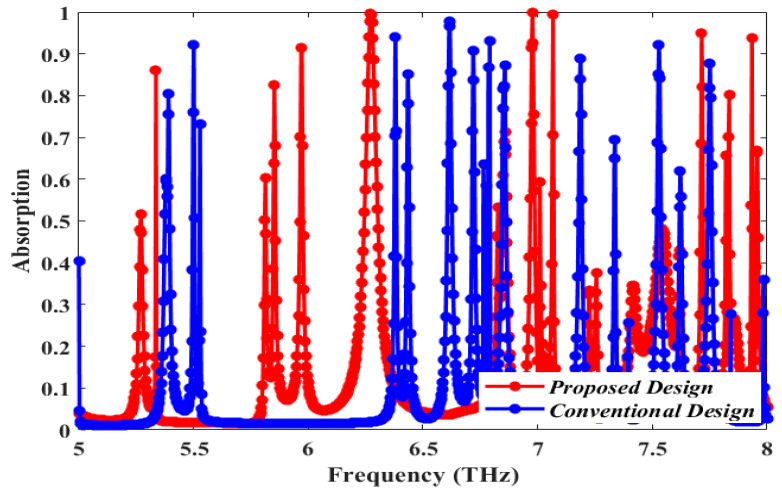
Comparison between the absorption plot of our proposed design and the conventional design (depicted in Figure 3) [31,32].

Simulated absorption plots of our proposed structure and the conventional one (depicted in Figure 3) are provided in Figure 4. A comparison between the two plots proves that the proposed design produces absorption peaks with enhanced absorbance compared to that of the conventional configuration. The proposed design has three absorption peaks above 95%, rising at 99.98%, 99.51%, and 99.33%. The conventional design presents a single peak above 95%, reaching an absorption of 97.79%. Thus, the convenience of our novel design is supported by a clear performance improvement with respect to conventional absorbers. The Quality Factor (Q-factor) is one of the key properties of MMA. It can be defined as follows:

(3) $$Quality\;Factor=\frac{Resonant\;Frequency}{FWHM}$$

**Figure 5:**
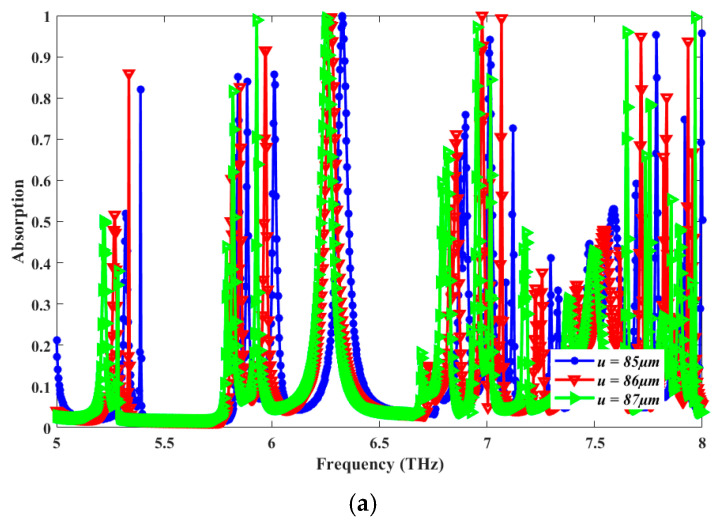

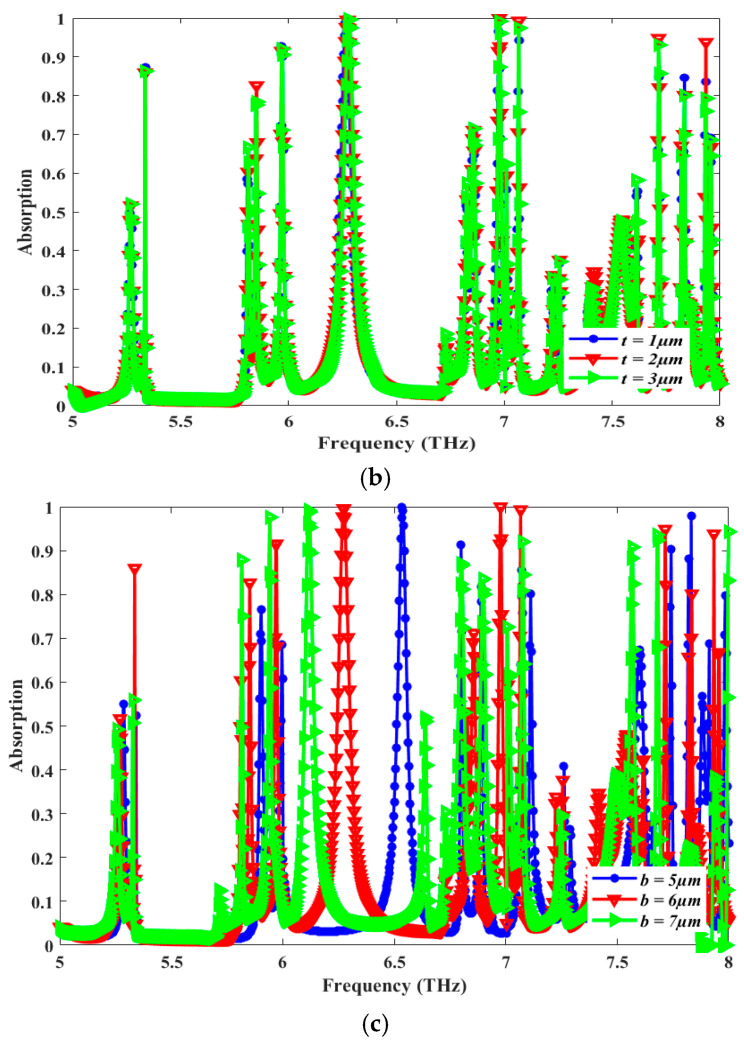
Results of parametric analysis plotting values of absorption A as a function of frequency for different values of unit cell dimensions *u* [µm] (**a**), ground plate thickness *t* [µm] (**b**), and height of the largest ring *b* [µm] (**c**).

In Figure 5, we report the results of the parametric-sweep simulation relevant to the main geometrical parameters of the proposed design used to target structure optimization. Figure 5a shows the results of the parametric sweep relevant to values of the unit cell dimension (*u*). The absorption spectra are plotted for different values of the parameter *u* ranging from 85 µm to 87 µm, with a step width of 1 µm. The results show that when the *u* parameter magnitude increases, the value of the resonant frequency decreases. Since u=86 µm attains the highest peak, it is chosen as the optimized unit cell dimension. It is observed that when the unit cell dimension is changed from its optimal value of 86 µm, there is a surface impedance mismatch, and impedance matching does not happen. This increases the magnitude of S_11,_ and the absorptivity reduces. It depicts that with an increase in the parameter magnitude, the resonant frequency decreases. Figure 5b shows the results of a parametric sweep relevant to values of the ground plate thickness (*t*) for three values of t from t=1 µm to t=3 µm with a step width of 1 µm. The spectra remain largely unchanged for all three considered plots. Hence, values of the *t* parameter may be leveraged as an additional degree of freedom in the design of the absorber’s ground plane in order to eliminate transmission. For this purpose, t=2 µm is chosen as an optimized value. Figure 5c shows the results of the parametric sweep relevant to values of the height of the outermost resonator from b=5 µm to b=7 µm with a step width of 1 µm. The resonant frequency gradually decreases as the parameter magnitude increases, but the best absorption is shown by  b=6 µm, chosen as the optimal value.

**Figure 6:**
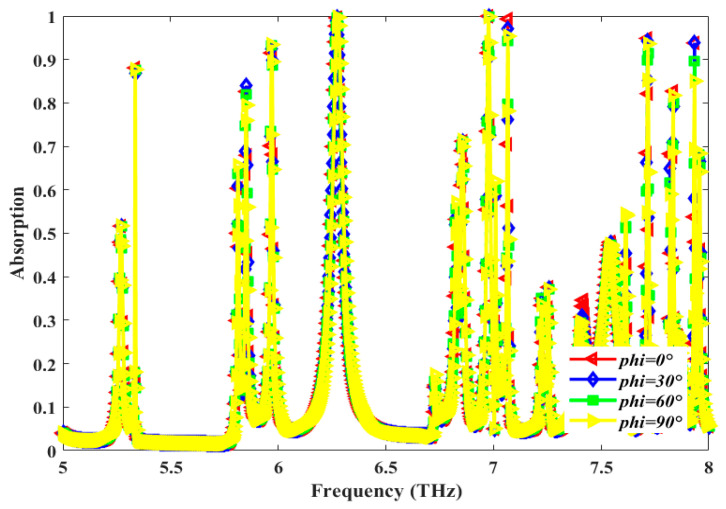
Plots of simulated absorption spectra for different values of the polarization angle (*ϕ*) [deg].

The numerical study of absorption spectra for different values of polarization angle, *ϕ*, is shown in Figure 6. Values of the angle *ϕ* vary from 0° to 90° with a step width of 30°. The absorption peaks and the resonance frequency remain largely unchanged when the polarization angle is varied, thereby making the proposed design polarization-insensitive in nature.

**Figure 7:**
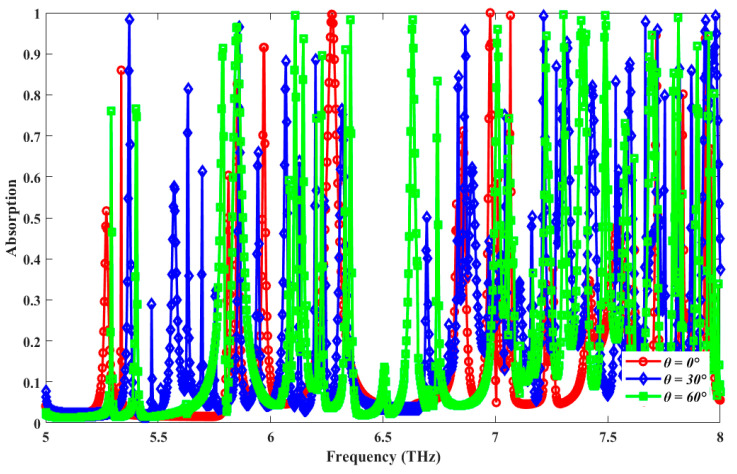
Plots of simulated absorption spectra for different values of incidence angles (*θ*) [deg].

Figure 7 shows plots of the absorption spectra for different values of the incidence angle (ϑ) ranging from 0° to 60° with a step width of 30°. It is evident that the absorption spectra are very stable even at a 60° angle of incidence, which is quite uncommon among reported all-metal absorbers at THz frequency in the recent literature.

**Figure 8:**
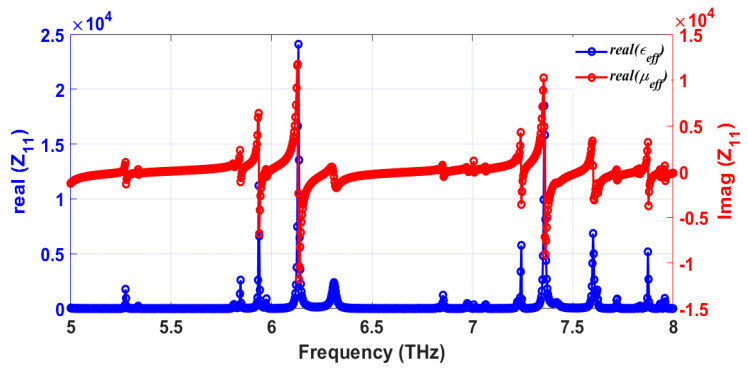
Real (blue solid line) and imaginary (red solid line) parts of the simulated impedance of the structure are plotted as a function of frequency.

**Figure 9:**
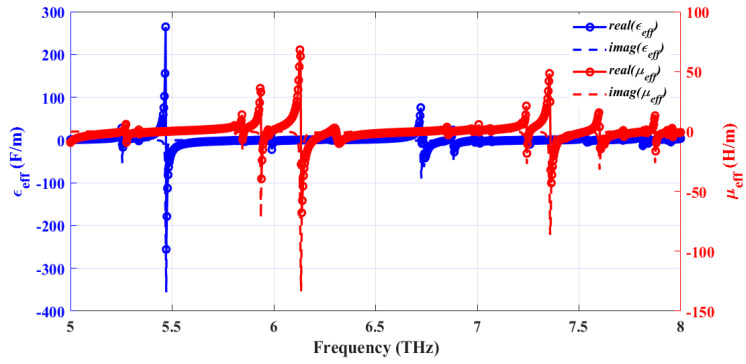
Simulated effective permittivity [F/m] (blue) and permeability [A/m] (red) of the structure are plotted as a function of frequency. The real and the imaginary parts are depicted in solid and dashed lines, respectively.

In Figure 8, real (blue solid line) and imaginary (red solid line) parts of the impedance Z_11_ of the simulated structure are plotted as a function of frequency. The results show that the real part of the effective impedance is close to 377 Ω, while the corresponding imaginary part approaches almost zero at each of the resonance frequencies; these values confirm impedance matching at resonance that assists in achieving such high absorption rates. Plots of the effective permittivity ε_eff_ and permeability µ_eff_ of the structure, shown in Figure 9, attest to the metamaterial nature of the proposed absorber. The proposed absorber exhibits both plasmonic and magnetic behaviors. A more detailed interpretation of the absorber’s behaviors at different resonating frequencies is summarized in Table 4.

The impedance, effective permeability, and effective permittivity of the structure can be calculated by using the formulae reported in the following equations:

(4) $$Z_{11}\left(f_{r}\right)=\sqrt{\frac{{\left(1+S_{11}\left(f_{r}\right)\right)}^{2}-S_{21}{\left(f_{r}\right)}^{2}}{{\left(1-S_{11}\left(f_{r}\right)\right)}^{2}-S_{21}{\left(f_{r}\right)}^{2}}}$$

(5) $$\varepsilon_{eff}\left(f_{r}\right)=\frac{c}{j\pi f_{r}d}\left(\frac{1-S_{21}-S_{11}}{1+S_{21}+S_{11}}\right)$$

(6) $${µ}_{eff}\left(f_{r}\right)=\frac{c}{j\pi f_{r}d}\left(\frac{1-S_{21}+S_{11}}{1+S_{21}-S_{11}}\right)$$

**Figure 10:**
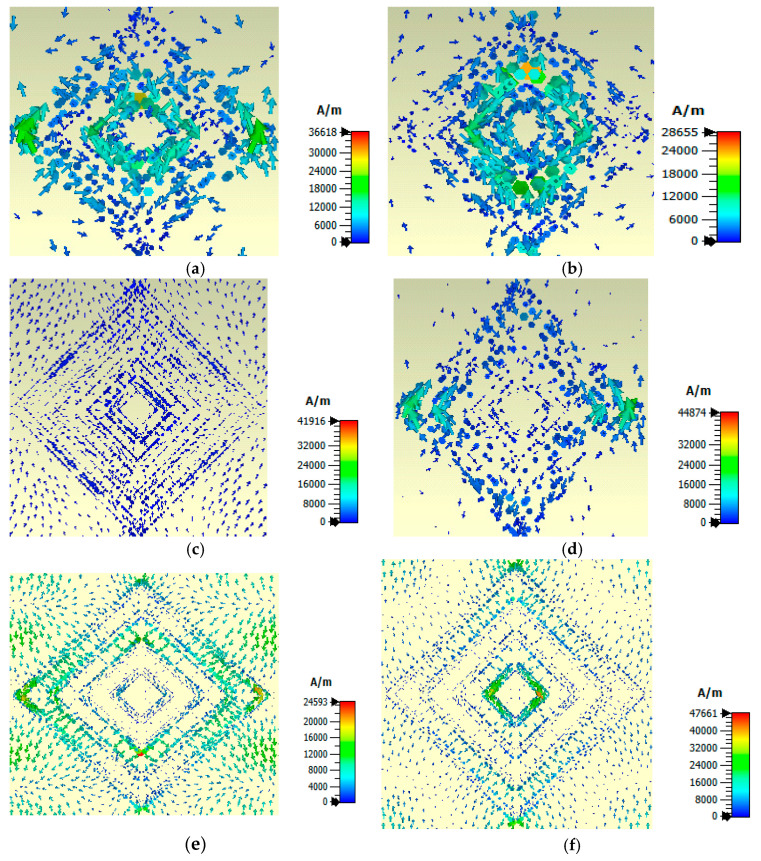
Simulated surface current distribution 2D map at the resonant frequency of (**a**) 5.972 THz, (**b**) 6.272 THz, (**c**) 6.977 THz, (**d**) 7.067 THz, (**e**) 7.715 THz, and (**f**) 7.934 THz.

According to the imposed periodic boundary conditions on the unit cell, the electric field is polarized along the *x*-axis, meaning that the *y*-*z* plane is a Perfect Electric Conductor with the presence of a nullified tangential component of the electric field, and the magnetic field is polarized along the *y*-axis, so the *x*-*z* plane is a Perfect Magnetic Conductor and similarly, the tangential component of the magnetic field becomes null. A plane wave traveling along the *z*-axis excites the unit cell: the incoming plane wave induces surface charge $J_{s}=n*H$, thereby making the magnetic field (*H*) discontinuous at the resonance, with ‘*n*’ being the unit normal vector to the plane of the absorber. Eventually, we obtain something close to a wire antenna with only oscillating surface currents, justifying the resonant nature of the metamaterial. From the surface current plot of Figure 10, six different patterns in six different resonating frequencies are observed. The first plot, Figure 10a, shows the maximum concentration of currents at the lateral edges of the outside resonators, relatively higher concentrations at the lateral edges of the inner resonators, and lower concentrations around the rest of the surface. Figure 10b shows maximum concentration around the longitudinal edges of the inner resonators, relatively higher concentration around the lateral edges of the inner resonators, and lower at the rest of the surface. Results shown in Figure 10c are different from those presented in Figure 10a,b,d: an almost uniform current distribution with a litter higher concentration around the longitudinal surface of the resonators is visible; current values are lower but uniform outside the last ring, and very low around the lateral edges of the resonators. Figure 10d represents a higher concentration around the lateral edges of the outside resonators, a slightly higher concentration around the longitudinal edges of the outside squares, but very little around the inner resonators and the rest of the surface. Figure 10e illustrates the maximum concentration along the lateral edges of the outer ring, the relatively higher concentration at the surface outside the resonators, and the lower concentration around the inside resonators. The highest concentration is visible at the corner of the second smallest ring. Figure 10f depicts the maximum concentration around the lateral edges of the smallest ring and longitudinal edges of the largest ring low concentration of current is seen at the rest of the surface.

**Figure 11:**
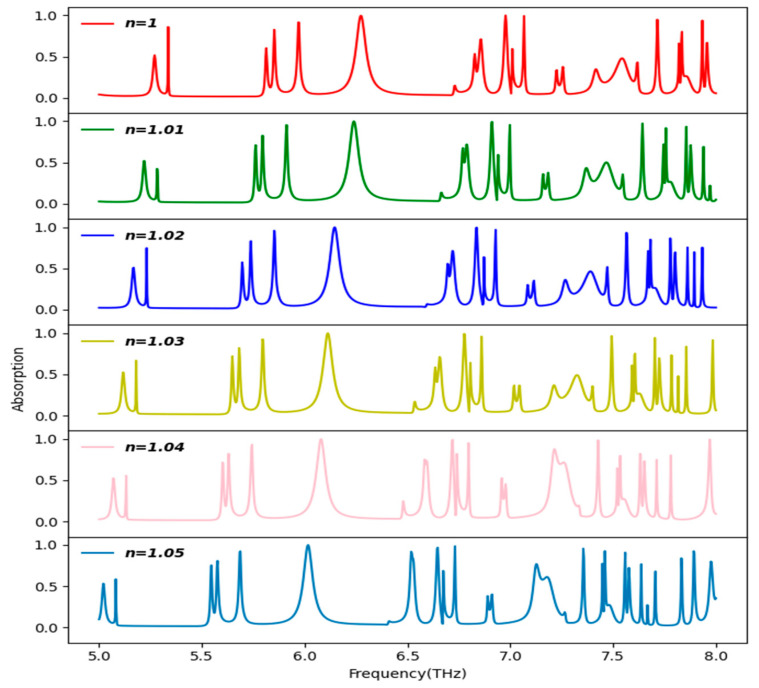
Shift in the absorption peaks in the absorption spectrum of the structure when the refractive index increases from 1 to 1.05.

The stacked plot of Figure 11 shows that when the refractive index of the surrounding medium is varied, a shift in the absorption spectrum is observed. In particular, when the refractive index is increased, a decrease or a left shift in the resonance frequency is observed. This inverse relationship allows the proposed absorber to be used as a refractive index sensor at THz frequency. By virtue of the fact that the THz frequency is non-ionizing in nature and is sensitive to weaker resonances, it allows the non-invasive probing of samples. In the figure, the absorption is plotted for different values of the refractive index in the range between 1 and 1.05 with a step size of 0.01. The refractive indices of many harmful gases lie in this range, as reported in Table 5 below [43].

**Figure 12:**
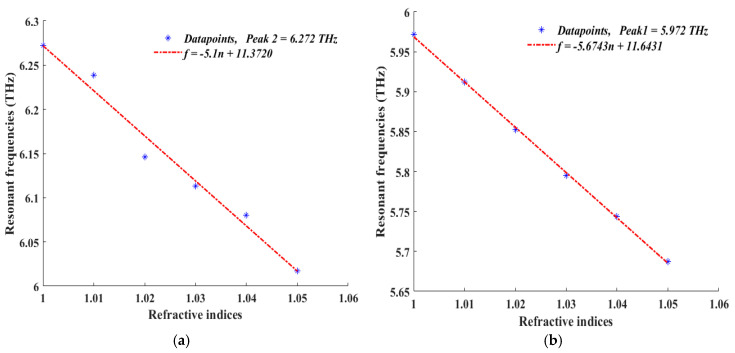

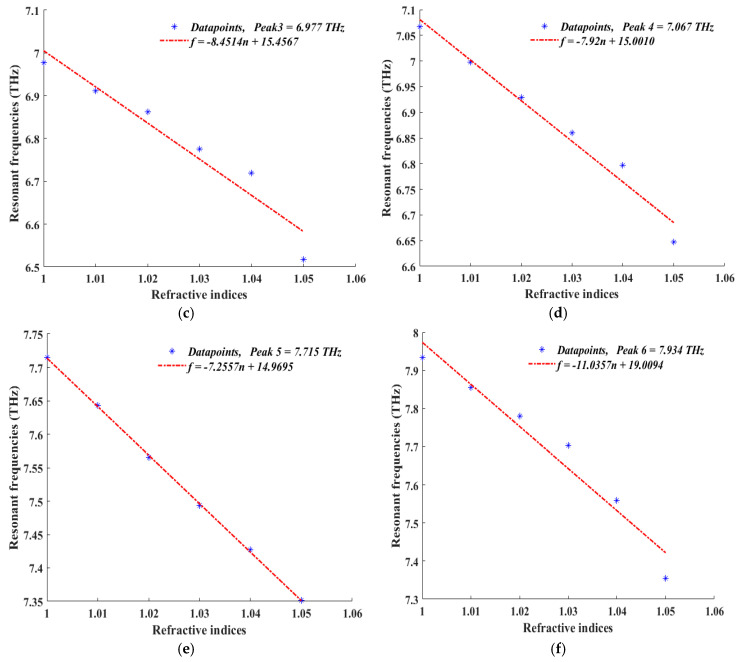
Scatter plots of resonance frequency with respect to values of surrounding medium refractive index in the range from 1 to 1.05 with a step width of 1.01 for each absorption peak; (**a**) 1st Peak = 5.972 THz, (**b**) 2nd Peak = 6.272 THz, (**c**) 3rd Peak = 6.977 THz, (**d**) 4th Peak = 7.067 THz, (**e**) 5th Peak = 7.715 THz, and (**f**) 6th Peak = 7.934 THz.

A sensor’s sensitivity is the most important performance metric; it can be expressed as the ratio of the resonance frequency shift to the refractive index alternations. We retrieve six distinct sensitivity plots and sensitivity values for the sensor design relevant to the six absorption peaks. The slope of the linear fit is computed in order to extract the sensitivity value from each plot. Figure 12a–f report scatter plots of values of each absorption peak at resonance versus values of the refractive index together with linear fits. The straight-line equations of the linear fits are reported in Equations (7)–(12):

(7) f=−5.67430n+11.6431

(8) f=−5.10000n+11.3720

(9) f=−8.45140n+15.4567

(10) f=−7.92000n+15.0010

(11) f=−7.25570n+14.9695

(12) f=−11.0357n+19.0094

The sensitivity, which is the slope of the curves, is calculated to be 5.67 THz/RIU, 5.1 THz/RIU, 8.45 THz/RIU, 7.92 THz/RIU, 7.25 THz/RIU, and 11.03 THz/RIU, respectively, for each peak, with an average of 7.57 THz/RIU. The calculated Figure of Merit (*FoM*) is 283.71, 85, 422.57, 792, 725.57, and 1103.57, respectively. *FoM* can be represented by this formula:

(13) $$FoM=\frac{Sensitivity(s)}{FWHM}$$

The sensitivity is, $s=\frac{∆f}{∆n}$; therefore, FoM can also be written as $FoM=\frac{∆f}{FWHM*∆n}$.

Now, $FWHM=f_{2}-f_{1}=∆f,$ substituting all the variables in Equation (13), we will obtain

(14) $$FoM=\frac{1}{∆n}$$

Since the value of the refractive index of air is 1, we can replace 1 with $\;n_{air}$. Thus, we can write *FoM* as

(15) $$FoM=\frac{n_{air}}{∆n}$$

Equation (15) links the sample’s refractive index changes to that of air to measurable quantities, providing the quantification of the results shown in Figure 11 and Figure 12 respectively.

## 4. Conclusions

In this paper, the design of an all-metal metamaterial-based sensor with novel geometry, which is composed of four square-ring resonators of different heights, is proposed. The distinctive feature of this design, consisting of the different heights of the square ring resonators, grants clear performance improvements with respect to conventional designs available in the literature. The proposed structure achieves six absorption peaks (three of which are characterized by more than 99% absorption) within a frequency range of 5 THz to 8 THz. In particular, absorption peaks and relevant frequencies are 91.52% at 5.972 THz, 99.6% at 6.272 THz, 99.98% at 6.977 THz, 99.34% at 7.067 THz, 94.85% at 7.715 THz, and 93.82% at 7.934 THz. The calculated sensitivity value for each of the six peaks is found to be 5.67 THz/RIU, 5.1 THz/RIU, 8.45 THz/RIU, 7.92 THz/RIU, 7.25 THz/RIU, and 11.03 THz/RIU. The average sensitivity amounts to 7.57 THz/RIU. The proposed structure proves to be suitably utilized as an all-metal metamaterial-based sensor demonstrating improved sensing capability and excellent performance and can be applied to biosensing and medical diagnostics at terahertz wavelengths. The respective Q-factors result to be 298.6, 104.63, 348.85, 706.7, 771.5, and 793.4.

Although the performed characterization confirms a multi-band behavior along with an enhancement in the sensing abilities of our metamaterial absorber, our study has been performed under normal incidence of the excitation wave; and for a particular angle of polarization. By applying machine-learning techniques, it is possible to generalize our results for any value of the above-mentioned angles, obtaining a complete computationally efficient evaluation of the structure’s performance. Metamaterial absorbers can also be employed in energy harvesting applications [45], especially solar energy at optical frequencies.

## Figures and Tables

**Table 1:** Parametric comparison of recent all-metal metamaterial sensors.

Ref No.	Peak Absorption (%)	No. of Absorption Peaks	Resonant Frequency (THz)	FWHM (THz)	Quality Factor	Sensitivity (THz/RIU)	Fig. of Merit (FOM)	Polarization-Insensitive
[35]	99.10	2	163.60	9.9750	16.400	197.49	12.79	Yes
[36]	99.05	2	1.9760	0.0108	181.61	0.971	57.42	Yes
[37]	99.85	4	297.45	7.3000	40.700	68.65	145.068	Yes
[38]	99.95	1	1.5630	0.0430	36.350	4.04	93.953	Yes
[39]	99.80	2	1.7230	0.0640	27.350	1.66	259.4	Yes
[40]	99.99	1	1.9900	0.0230	87.000	0.54	23.5	No
[32]	99.99	1	0.4978	0.00026	1920.0	0.32	1230	Yes
This paper	91.52, 99.60, 99.98, 99.34, 94.85, 93.82	6	5.972, 6.272, 6.977, 7.067, 7.715, 7.934	0.02, 0.06, 0.02, 0.01, 0.01, 0.01	298.60, 104.53, 348.85, 706.70, 771.5, 793.4	5.6743 5.1 8.4514 7.92 7.2557 11.0357	283.715, 85, 422.57, 792, 725.57, 1103.57	Yes

**Table 2:** Parameter list of the proposed design.

Parameter Definition	Parameter Symbol	Measure (µm)
Unit cell periodicity	u	86
Ground plate thickness	t	2
First ring height	b	6
Radius of first ring	r	40
Radius of second ring	r_1_	30
Radius of third ring	r_2_	20
Radius of last ring	r_3_	10
Ring thickness	*a*	2

**Table 3:** The calculated parameters of our proposed design.

Resonant Frequency (THz)	Peak Absorption (%)	FWHM (THz)	Quality Factor	Sensitivity (THz/RIU)	Figure of Merit (FOM)
5.972	91.52	0.02	298.60	5.6743	283.715
6.272	99.60	0.06	104.63	5.1	85
6.977	99.98	0.02	348.85	8.4514	422.57
7.067	99.34	0.01	706.7	7.92	792
7.715	94.85	0.01	771.5	7.2557	725.57
7.934	93.82	0.01	793.4	11.0357	1103.57

**Table 4:** Qualitative and quantitative descriptions achieved by resonance.

Resonant Frequency (THz)	Impedance, Z (Ω)	Permittivity ε (F/m)	Permeability µ (H/m)	Absolute Value of Impedance	Type of Metamaterial Response	Type of Resonance
5.972	477.357 + j218.392	Negative	Almost zero	524.94	Epsilon-Negative	Plasmonic
6.272	283.316 − j 89.7128	Positive	Negative	297.18	Mu-Negative	Magnetic
6.977	438.297 + j57.8342	Negative	Almost zero	442.09	Epsilon-Negative	Plasmonic
7.067	358.531 + j30.1279	Negative	Almost zero	359.79	Epsilon-Negative	Plasmonic
7.715	230.387 + j58.1584	Negative	Almost zero	237.61	Epsilon-Negative	Plasmonic
7.934	408.552 − j193.915	Almost zero	Almost zero	452.23	Epsilon-Zero	Plasmonic

**Table 5:** Refractive indices of harmful gases.

Harmful Gas	Refractive Index	Harms Caused
Benzene	1.001762	Skin, Eye Irritation
Chloroform	1.001450	Unconscious
Chlorine	1.000773	Skin, Eye Irritation
Carbon disulfide	1.001481	Severe skin and eye irritation
Ether, methyl	1.000891	Severe skin and eye irritation
Carbon dioxide	1.00045	Headache, dizziness
Carbon monoxide	1.0003364	Fatigue, Headache

## Data Availability

Data are contained within the article.

## Abbreviations

The following abbreviations are used in this manuscript:

MMA	Metamaterial Absorber
RIU	Refractive Index Unit
FWHM	Full Width Half Maximum
FoM	Figure of Merit
Q-factor	Quality Factor
m-MIMO	massive multiple-input multiple-output
